# Central Retinal Vein Occlusion in a Young Woman with Diabetes and Hypertension after mRNA-Based COVID-19 Vaccination—A Case Report and Brief Review of the Literature

**DOI:** 10.3390/vaccines11020365

**Published:** 2023-02-05

**Authors:** Shao-Yu Sung, Laura A. Jenny, Yo-Chen Chang, Nan-Kai Wang, Pei-Kang Liu

**Affiliations:** 1 Department of Ophthalmology, Kaohsiung Medical University Hospital, Kaohsiung Medical University, Kaohsiung 807, Taiwan; 2 School of Medicine, College of Medicine, Kaohsiung Medical University, Kaohsiung 807, Taiwan; 3 Department of Ophthalmology, Edward S. Harkness Eye Institute, Columbia University Irving Medical Center, New York, NY 10032, USA

**Keywords:** central retinal vein occlusion, COVID-19, COVID-19 vaccination

## Abstract

A 25-year-old female with diabetes and hypertension presented with progressive painless blurred vision in her left eye ten days after she received her third dose of the SARS-CoV-2 mRNA vaccine BNT162b2 (Pfizer-BioNTech). The clinical examination confirmed the diagnosis of Central Retinal Vein Occlusion (CRVO) complicated with macular edema. Three doses of anti-vascular endothelial growth factor (VEGF) were injected intravitreally. Visual acuity was improved from 20/100 to 20/30, but recurrence was noted at 6 months. Several cases of retinal vein occlusion (RVO) after COVID-19 vaccination have been reported. However, the present case is the youngest female individual documented to have CRVO after SARS-CoV-2 vaccination. This case demonstrates that the macular edema might be recurrent in patients with risk factors for CRVO who receive SARS-CoV-2 vaccination, suggesting the need for careful consideration of the treatment strategy and close follow-up. Although the definite pathogenesis still needs to be carefully determined, this report highlights the possible association between RVO and mRNA-based COVID-19 vaccination, even in young individuals.

## 1. Introduction

Given the threat of the coronavirus disease 2019 (COVID-19) caused by the severe acute respiratory syndrome coronavirus 2 (SARS-CoV-2) infection worldwide, there has been vigorous research for effective treatment strategies and vaccine development to contain the pandemic. Vaccines help protect against certain diseases by providing individual and herd immunity, serving an important role in global public health. The four categories of SARS-CoV-2 vaccines include mRNA vaccines, which deliver genetic code through lipid nanoparticles (e.g., BNT162b2, Pfizer-BioN-Tech [1], mRNA-1273 and Moderna [2]), protein subunit vaccines, which extract short fragments of proteins from inactivated disease-causing microorganisms (e.g., NVX-CoV2373, Novavax [3]), vector vaccines, which use viral carriers to deliver DNA signal sequences of spike proteins (e.g., Ad26COVS1, Janssen Johnson & Johnson [4], AZD1222 and Oxford-AstraZeneca [5]) and whole virus vaccines (e.g., PiCoVacc, Sinovac, BBIBP-CorV and Sinopharm). Eleven vaccines were granted emergency use listing (EUL) by the World Health Organization (WHO) [6]. Out of these eleven vaccines, Pfizer-BioN-Tech, Moderna, Oxford-AstraZeneca and Janssen Johnson & Johnson were approved by most countries worldwide and thus are more commonly used.

The reported side effects of COVID-19 vaccination include thrombosis with thrombocytopenia, Guillain–Barre syndrome and myocarditis. In addition, several ocular adverse effects after COVID-19 vaccination have been reported [7]. Here, we present the case of a 25-year-old woman who developed central retinal vein occlusion (CRVO) after BNT162b2 administration. We also describe retinal vein occlusion (RVO) after COVID-19 vaccination and outline key steps for providers to take to monitor patients after vaccination. To date, this report details the youngest female patient who developed CRVO after receiving BNT162b2.

## 2. Case Report

**Figure 1:**
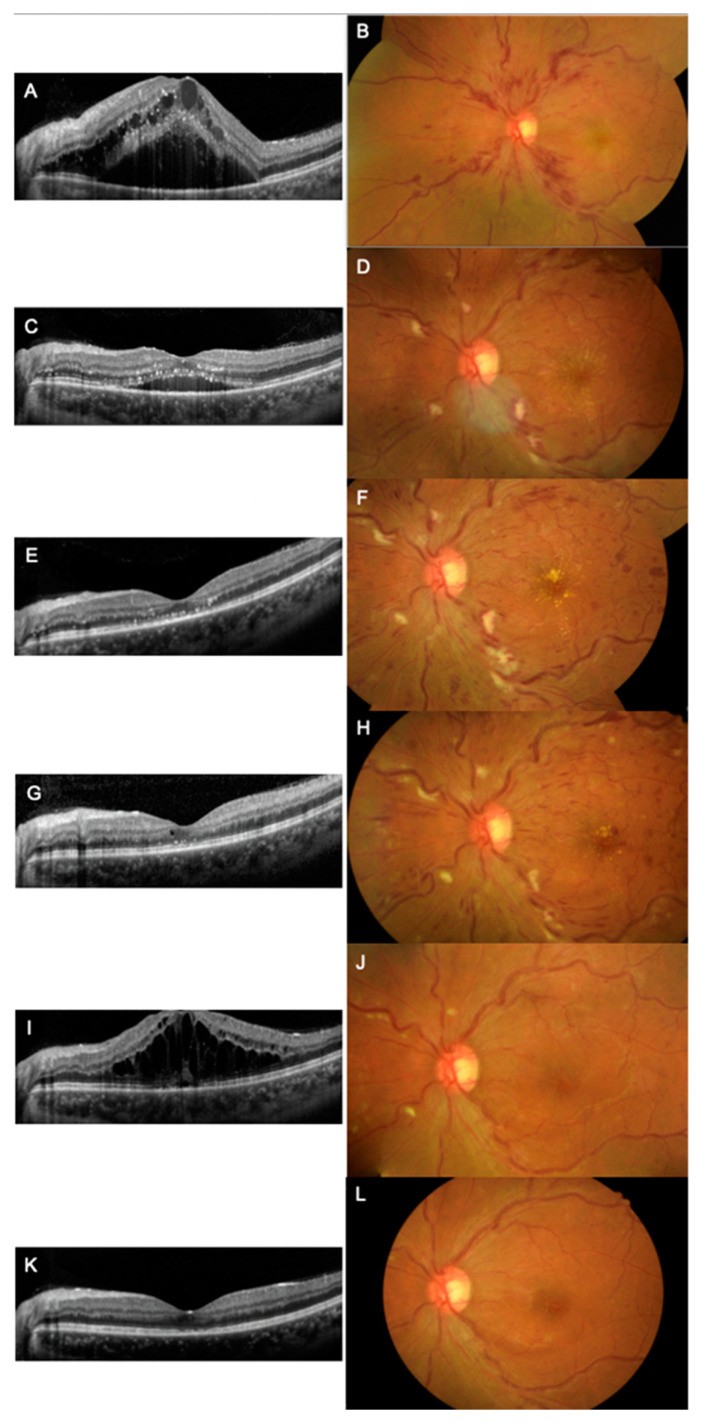
SD-OCT and color fundus imaging over the 7 months since first presentation. (**A**,**B**) Initial presentation of diffuse flame shape hemorrhage, macular edema and disc edema with visual acuity of 20/100 in the left eye. (**C**,**D**) Three weeks after intravitreal injection of ranibizumab, macular edema decreased. Intravitreal injection of aflibercept was given for incomplete treatment response to ranibizumab. (**E**,**F**) Six weeks after the diagnosis, macular edema subsided. (**G**,**H**) Three months after the diagnosis, progressive retinal hemorrhage was observed. A second dose of aflibercept was given. (**I**,**J**) Six months after the initial diagnosis, recurrent macular edema was observed and the third dose of aflibercept was administered. (**K**,**L**) Seven months after the diagnosis, macular edema completely subsided with no retinal hemorrhage observed.

**Figure 2:**
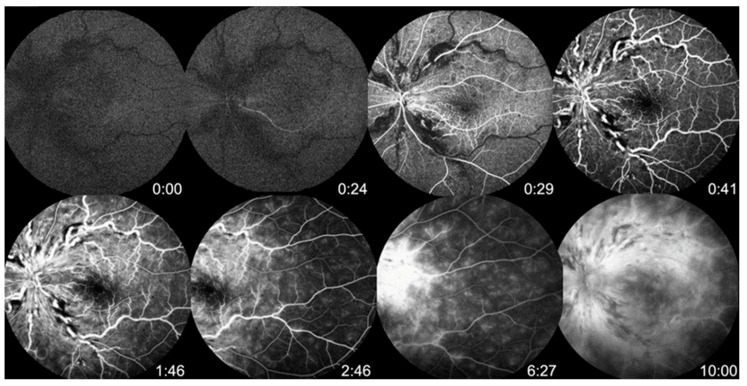
Fluorescein angiography (FA) of the left eye showed delayed arterial phase and arteriovenous transit time without significant ischemic areas in the retina.

**Figure 3:**
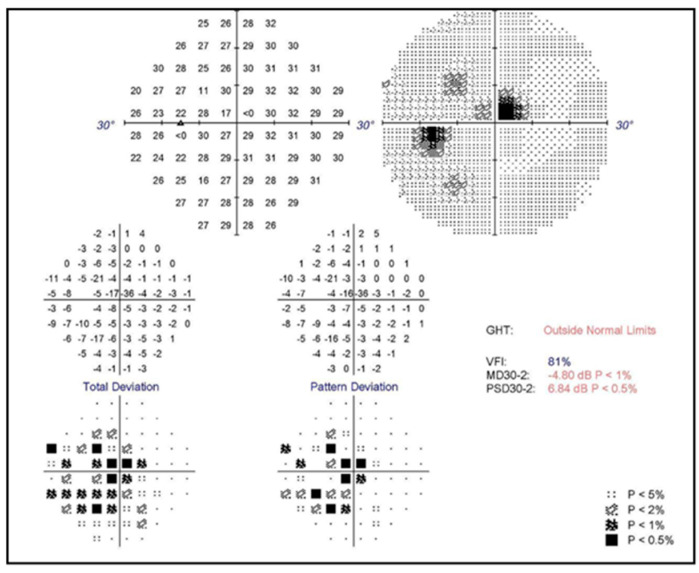
Auto-perimetry (Humphrey 30*−*2) of the left eye. A paracentral scotoma with mean deviation of −4.80 dB was noted on the left eye.

A 25-year-old obese female with a history of hypertension managed by regular oral medications presented with progressive, painless blurred vision in her left eye for several days. Ten days before symptom onset, she received her third dose of the SARS-CoV-2 mRNA vaccine BNT162b2 (Pfizer-BioNTech). She did not have a history of taking oral contraceptive pills or a history of smoking. She had no history of allergies. At presentation, the best corrected visual acuity was 20/100 in the left eye and 20/20 in the right eye. Intraocular pressure was 13 mmHg in the right eye and 18 mmHg in the left eye. No relative afferent pupil defect was found. Slit lamp anterior segment examination showed no abnormal findings. In the left eye, fundus examination revealed a diffuse flame shape hemorrhage, disc edema and severe macula edema (Figure 1A,B). Fluorescein angiography (FA) disclosed delayed arterial phase and arteriovenous transit time without significant ischemic areas in the retina (Figure 2). Auto-perimetry showed paracentral scotoma (Figure 3). Spectral domain optical coherence tomography (SD-OCT) revealed severe subfoveal fluid accumulation. A thorough survey of laboratory examinations was completed (Table 1). Aside from an incident finding of newly diagnosed type 2 diabetes mellitus (HbA1c: 9.2%), lab data associated with hyperlipidemia and hypercoagulopathy were within normal limits. Carotid ultrasound imaging revealed normal findings. Due to severe macular edema, she received an intravitreal injection of ranibizumab (IVR). Three weeks later, there was sustained subfoveal fluid, although the total amount of fluid had decreased from the first visit (Figure 1C). The patient was given intravitreal injections of aflibercept (IVA) four weeks after receiving the IVR. Six weeks after initial presentation, the macular edema completely subsided. SD-OCT showed decreased central macular thickness (Figure 1E) and her best corrected visual acuity was improved to 20/40. However, progressive retinal hemorrhage was noted 12 weeks after diagnosis of the disease (Figure 1H). She then received a second dose of IVA. The visual acuity further improved to 20/32 shortly after injection. Six months after the diagnosis, SD-OCT revealed recurrent macular edema (Figure 1I), and a third dose of IVA was then administered. Macular edema was completely absent and no new retinal hemorrhage was found at her last visit (7 months after diagnosis) (Figure 1K,L).

## 3. Discussion

Although rare, several ocular manifestations after SARS-CoV-2 vaccination have been documented. Graves’ disease activation, corneal graft rejection, herpes zoster ophthalmicus (HZO), uveitis, Vogt–Koyanagi–Harada (VKH) disease, acute abducens nerve palsy, Bell’s palsy, arteritic anterior ischemic optic neuropathy, central serous chorioretinopathy, RVO and cerebral venous sinus thrombosis have been observed in patients who received the Pfizer-BioN-Tech vaccine [7,8,9,10]. Bilateral immune-mediated keratolysis, corneal graft rejection, VKH disease, multifocal choroiditis, acute macular neuroretinopathy (AMN), optic neuritis, superior ophthalmic vein thrombosis and cerebral venous sinus thrombosis have been associated with the Oxford-AstraZeneca vaccine [7,8,9,10]. No causal relationships between vaccinations and the diseases mentioned above have been established to date. With the growing number of documented complications after vaccination and the potential ocular adverse events following COVID-19 vaccination, physicians should carefully follow patients after vaccination.

Retinal vein occlusion, commonly seen in the ophthalmologic clinical practice, is the second leading cause of retinal vascular blindness after diabetic retinopathy. It is classified into branch RVO (BRVO), hemiretinal vein occlusion (HRVO) and CRVO based on the location of the obstruction. The typical fundus findings associated with CRVO include tortuosity and dilatation of all branches of the central retinal vein, dot/blot and flame-shaped hemorrhages, optic disc and macula edema and cotton-wool spots. CRVO is further categorized into the ischemic and non-ischemic types based on the perfusion status. Ischemic CRVO tends to present with poor visual acuity, the presence of relative afferent pupillary defects, non-perfusion of more than 10 disc areas on FA and a higher probability of negative ERG patterns. Venous compression at arteriovenous crossings, degenerative venous change including venous stasis and endothelial damage along with hypercoagulable states may be involved in the pathogenesis. The likelihood of developing RVO increases with age [11]. A total of 90% of cases occur in patients over the age of 55 years. Common atherosclerosis risk factors such as hypertension, diabetes, hyperlipidemia and smoking are commonly reported to be associated with RVO. Less commonly, blood coagulation disorders and systemic inflammatory disorders are also risk factors of RVO [12,13]. According to The Beaver Dam Eye Study, a population-based study, the prevalence of CRVO and BRVO were 0.1% and 0.6%, respectively. The population included indivisuals that were 43 to 84 years of age. The odds ratio of hypertension and diabetes were 5.42 and 2.43 for the incidence of BRVO, respectively [14]. From a review summarizing studies in the Unites States, Europe, Asia and Australia, with 68751 individuals with ages ranging from 30 to 101 years, the prevalence of any RVO, BRVO and CRVO were 0.52%, 0.442% and 0.08%, respectively. Given the extremely low prevalence in young people, data for those under 30 years of age are not available in these reports. In the group aged 30 to 39 years, the prevalence of any RVO, BRVO and CRVO were 0.162%, 0.162% and 0%, respectively [15]. These studies showed that younger individuals, especially those under the age of 30, rarely experience RVO.

A total of 22 papers with 51 cases that present with RVO after COVID-19 vaccination are summarized in Table 2. Some cases had risk factors of hypertension, diabetes mellitus and hyperlipidemia, but some cases did not describe in detail the concomitants of other systemic diseases. There were 21 cases of CRVO (Table 3). Of the 21 cases, 1 of them included findings of central retinal artery occlusion (CRAO), 1 included findings of CRAO and ischemic optic neuropathy and a third included findings of CRAO and exudative retinal detachment. There were 13 males and 8 females, ranging in age from 13 to 96 years old. Nine of them received BNT162b2, nine received AZD1222, and single cases received mRNA-1273, Ad26.COV2 and Corbevax, respectively. Symptoms appeared between 15 min and 28 days after vaccination. There were 26 cases of RVO after BNT162b2 vaccination (Table 4). A total of 9 of them were cases of CRVO, 13 of them were cases of BRVO, 1 of them was a case of HRVO and 5 of them did not specify the RVO type. Male cases were more predominant over female cases in these three groups. In addition, the average age was 57 (ranging from 27 to 96 years old), and the other two groups had an average age of 65 and 79 years old, respectively. Of the 8 cases of CRVO after BNT162b2, only 1 case of a 96-year-old had risk factors of HTN and DM. According to previous reports, the side effects of the vaccine do not necessarily occur after the first dose. A total of 22 of the 51 cases from the published reports presented with RVO after the 2nd dose of the vaccination [16,17,18,19,20,21,22,23,24,25,26,27,28]. There was 1 case which showed symptoms appearing 25 days after receiving the 3rd dose of AZD1222 [29].

Recurrent retinal hemorrhage and macular edema occurred in the present case after three doses of intravitreal injection in a six-month follow-up period while the patient’s systemic diseases were well-managed. Most published cases have relatively short periods of follow-up time and lack presentation of the patients’ final visual acuities. There are three cases of CRVO after BNT162b2 with initial VA of 20/30, 20/40 and 20/40, who had VA of 20/20 after IVI anti-VEGF without detailed follow-up information [16,31,34]. Our case demonstrates that macular edema caused by vaccine-associated CRVO may be recurrent, and may cause poor initial visual acuity.

Based on the Naranjo scoresheet for assessing the treatment-adverse reaction association [38], the subject scored 5 points, indicating a probable association. However, since the complications of CRVO are often irreversible and further serious complications such as neovascular glaucoma may happen, documentation of potential association is important.

The early onset of CRVO at 25 years of age indicates that other mechanisms associated with hypercoagulability and inflammatory processes need to be considered. For this rare ocular complication associated with vaccination, it is difficult to confirm a causal relationship. Findings from previous case reports, and the young age of the patient, allow for the supposition that vaccination may have played a role in CRVO development. Vaccines for COVID-19 were designed to produce high levels of neutralizing antibodies targeting spike proteins and to activate T-helper responses. Several possible mechanisms of adverse reactions to vaccination have been described, including autoimmune/inflammatory syndrome induced by adjuvants (ASIA), altered metabolism of RNA (spike protein) in susceptible individuals, stimulation of innate immunity through nucleic acid receptors (e.g., TLRs), molecular mimicry between vaccine peptide fragments and human-derived proteins and delayed hypersensitivity with deposition of immune complexes [7,8]. When it comes to the thrombotic events associated with BNT162b2 vaccines, vasculitis or hypercoagulable status caused by antigen-specific cell- and antibody-mediated hypersensitivity reactions and molecular mimicry might be the possible mechanism. Moreover, PF4 antibodies, mainly described in thrombosis with thrombocytopenia syndrome (TTS), have also been found in the patients receiving BNT162b2 vaccines [39]. In spite of the hypothesis mentioned above, the direct association of COVID-19 vaccination with RVO requires further study to clarify the pathogenesis.

## 4. Conclusions

To date, the present case is the youngest (25 years old) female individual who presented with CRVO after COVID-19 vaccination. However, definite causality needs to be established carefully. Physicians should closely monitor patients for ocular adverse effects following COVID-19 vaccination, and this report provides information on potential treatments for approaching similar complications. Patients with risk factors for RVO, even those who are young, should be closely followed for complications after vaccination. Overall, the incidence of CRVO following COVID-19 vaccination appears to be low. The benefits of vaccinations still outweigh the potential risks, but patients should receive prompt ocular examination when they notice minor visual impairments after vaccination.

## Figures and Tables

**Table 1:** Clinical laboratory results of the patient. Except for HbA1c*, other data were negative or within normal limits.

Laboratory Test	Results	Laboratory Test	Results
White Blood Cell(/μL)	8350	RPR	Non-Reactive
Urea N (mg/dL)	7.6	ANA	<1.40
Creatinine (mg/dL)	0.48	RA factor (IU/mL)	<10.0
Na (m mol/L)	134	PT (second)	10.5
K (m mol/L)	3.7	PTT (second)	27.1
SGOT (IU/L)	18	D-Dimer (mg/L FEU)	0.19
SGPT (IU/L)	27	Protein C (%)	97
CRP (mg/L)	1.98	Protein S (%)	88
ESR (mm/h)	9	Homocysteine (μmol/L)	6.59
*HbA1c (%)	9.2	Anti-cardiolipin antibody	negative
Cholesterol (mg/dL)	148	Anti-β2 glycoprotein-I antibody	negative
TG (mg/dL)	8	LAC (lupus anticoagulant) (ratio)	<1.2

**Table 2:** Summary of studies that describe retinal vein occlusion (RVO) after COVID-19 vaccination.

Study	Case	Vaccine	First Onset (day)	Age	Sex	Risk Factors	Diagnosis	Management	Visual Acuity (Initial→Final)
**Priluck et al.** **(Apr. 22)** [17]	1	2nd mRNA-1273	-	57	F	HTN	BRVO	Aflibercept monthly for 6 months	20/30->20/25
**Tanaka et al.** **(Nov. 21)** [18]	2	2nd BNT162b2	1 R	71	F	History of BRVO	BRVO	Aflibercept*1	20/30->20/20
		1st BNT162b2	1 R	72	M	History of BRVO	BRVO	Ranibizumab*2	20/25->20/25
**Peters et al.** **(Jan. 22)** [19]	5	1st AZD1222	2	71	M	Unremarkable	BRVO	Bevacizumab (monthly)	6/60
		1st AZD1222	3	58	M	Unremarkable	HRVO	Bevacizumab (monthly)	6/18
		1st AZD1222	3	73	F	HTN	BRVO	Aflibercept (monthly)	6/19
		1st BNT162b2	5	47	F	Unremarkable	BRVO	Bevacizumab (monthly)	6/9.6
		2nd BNT162b2	1–3	36	M	Unremarkable	CRVO	Aflibercept (monthly)	6/9
**Sugihara et al. (Jan. 22)** [20]	1	2nd BNT162b2	11 L	38	M	Unremarkable	BRVO	Aflibercept*2	0.9->1.2
**Pur et al.** **(Feb. 22)** [30]	1	1st BNT162b2	2 R	34	M	Unremarkable	BRVO	None	20/20
**Endo et al. (Sep. 21)** [31]	1	1st BNT162b2	15 L	52	M	Unremarkable	CRVO	Steroid*1 Bevacizumab*1	20/30->20/20
**Ikegami et al.(Nov. 2021)** [21]	1	2nd mRNA-1273	2 R	54	F	Unremarkable	CRVO + CRAO	None	NLP
**Sacconi et al. (Dec. 2021)** [22]	1	2nd mRNA-1273	2 R	74	F	Unremarkable	HRVO	Ranibizumab*2	20/40->20/32
**Bialasiewicz et al. (Aug. 2021)** [16]	1	2nd BNT162b2	15 min L	50	M	Unremarkable	CRVO	Aflibercept	0.5->1.0
**Park et al.** **(Dec. 2021)** [23]	11	1st AZD1222	1 L	68	F	Hyperlipidemia	RVO	None	HM
		1st BNT162b2	2 L	76	M	HTN	BRVO	None	0.8
		2nd BNT162b2	1 R	85	F	DM, HTN	RVO	Bevacizumab	CF10
		1st AZD1222	2 L	59	M	DM, HTN	RVO	None	0.8
		1st AZD1222	2 R	61	M	Unremarkable	CRVO	Bevacizumab	0.04
		2nd BNT162b2	2 L	79	M	DM	RVO	Bevacizumab	0.04
		1st BNT162b2	16 L	77	F	HTN	BRVO	Bevacizumab	0.8
		1st BNT162b2	13 R	63	M	DM	RVO	Bevacizumab	0.01
		1st AZD1222	21 L	51	F	HTN	BRVO	Bevacizumab	0.09
		1st BNT162b2	4 L	81	F	HTN	RVO	None	0.3
		1st AZD1222	3 L	61	M	HTN	CRVO	None	0.9
**Sonawane et al.** **(Dec. 2021)** [24]	2	2nd AZD1222	4 R	50	M	DM	CRVO	Anti-VEGF	6/60
		2nd AZD1222	3 R	43	F	Unremarkable	CRVO	None	5/60
**Shah et al.** **(Dec. 2021)** [32]	1	1st BNT162b2 Worsen after 2nd	Few days	27	F	Unremarkable	CRVO	Ranibizumab	improved
**Lee et al.** **(Jan. 2022)** [25]	1	2nd BNT162b2	10–12 L	34	M	Unremarkable	CRVO + CRAO + ischemic optic neuropathy	Aflibercept anti-inflammatories, diuretics, anti- platelet agents and anticoagulant	CF->20/30
**Sodhi et al.** **(June 2022)** [33]	1	1st AZD1222	3 L	43	M	Unremarkable	CRVO	Triamcinolone *1	20/630->20/200
**Romano et al.** **(July 2022)** [26]	1	2nd AZD1222	2 R	54	F	HTN	CRVO	Dexamethasone implant + PRP	20/400 ->20/200
**Takacs et al.** **(Aug 2022)** [34]	1	1st BNT162b2	14 R	35	M	Unremarkable	CRVO	Aflibercept Oral aspirin	0.5 ->1.0
**Karageorgiou et al.** **(Sep 2022)** [35]	1	AZD1222	7 R	60	M	Unremarkable	BRVO	Anti-VEGF	-
**Nangia et al.** **(Sep 2022)** [36]	1	1st Corbevax	28 L	13	M	Unremarkable	CRVO	IVMP 0.5 g for 3 days followed by oral steroid	6/7.5->6/6
**Majumder et al.** **(May 2022)** [29]	1	3rd AZD1222	25 R	28	M	Unremarkable	CRVO	IVMP 1 g for 3 days followed by oral steroid	2/60->6/9
**Chen et al.** **(July 2022)** [27]	1	2nd BNT162b2	10 R	72	M	Unremarkable	CRVO + CRAO + exudative RD	Aflibercept IVMP 1 g for 3 days followed by oral steroid PRP	HM->20/400
**Vujosevic et al.** **(May 2022)** [28]	13	1st AZD1222	7 R	69	F	(Deep venous thrombosis)	BRVO	Laser photocoagulation	20/32->20/20
		2nd BNT162b2	14 R	82	F	Unremarkable	BRVO	Steroid treatment	20/63->20/40
		2nd BNT162b2	7 R	96	F	HTN, DM	CRVO	Steroid treatment	20/200->20/200
		2nd BNT162b2	10 L	91	F	Unremarkable	CRVO	None	CF
		2nd BNT162b2	7 both	78	F	Unremarkable	BRVO	Anti-VEGF, R	20/25->20/20(R) 20/20(L)
		1st AZD1222	7 R	70	M	Unremarkable	CRVO	None	20/20
		1st AZD1222	14 R	40	M	Hyperhomocysteinemia	BRVO	None	20/20
		2nd BNT162b2	28 R	91	M	DM	BRVO	Steroid treatment	20/32->20/32
		2nd BNT162b2	21 R	72	F	HTN hyperlipidemia	BRVO	Steroid treatment	20/25->20/20
		2nd BNT162b2	14 R	88	M	HTN, hyperlipidema	HRVO	Steroid treatment	20/125->20/125
		2nd AZD1222	28 R	73	F	HTN, hyperlipidemia	CRVO	Steroid treatment	CF->CF
		1st Ad26. COV2	7 R	65	F	HTN, hyperlipidemia, DM	CRVO	Steroid treatment	20/40->20/32
		1st AZD1222	14 L	72	F	HTN	HRVO	Laser photocoagulation	20/50->20/50
**Tanaka et al.** **(May 2022)** [37]	2	1st BNT162b2	3 R	50	F	Unremarkable	BRVO	Ranibizumab*3	20/25->20/20
		1st BNT162b2	3 R	56	F	Unremarkable	BRVO	Ranibizumab*3	13/20->20/20

Abbreviations: HTN: hypertension, F: female, M: male, R: right eye, L: left eye, both: both eyes, BRVO: branch retinal vein occlusion, CRVO: central retinal vein occlusion, CRAO: central retinal artery occlusion, HRVO: hemi retinal vein occlusion, NLP: no light perception, HM: hand motion, CF: counting finger, DM: diabetes mellitus, Anti-VEGF: anti-vascular endothelial growth factor, PRP: panretinal photocoagulation, IVMP: intravenous methylprednisolone.

**Table 3:** Twenty-one cases of published central retinal vein occlusion (CRVO) after COVID-19 vaccination.

**Diagnosis**	CRVO: 18 CRVO + CRAO: 1(mRNA-1273) CRVO + CRAO + ischemic ON: 1(BNT162b2) CRVO + CRAO + exudative RD: 1 (BNT162b2)
**Sex**	Male: 13 Female: 8
**Age**	13–96
**Vaccine**	BNT162b2: 9 AZD1222: 9 mRNA-1273: 1 Ad26. COV2: 1 Corbevax: 1
**Onset after vaccination**	15 min to 28 days
**Risk factors**	HTN: 5 DM: 3 Hyperlipidemia: 1
**Treatment**	IVI anti-VEGF (Aflibercept, Ranibizumab, Bevacizumab) and steroid, IVMP, laser photocoagulation

**Table 4:** Twenty-six cases of RVO after BNT162b2 vaccination.

	CRVO	BRVO	RVO
Cases	8	13	5
Sex (Male/Female)	5/3	7/6	3/2
Age	27–96(Average: 57)	34–91(Average: 65)	63–88(Average: 79)
Risk factors	1 96 y/o patient and HTN and DM	HTN: 6 DM: 1 (deep vein thrombosis): 1 Hyperlipidemia: 1 Hyperhomocysteinemia: 1	DM:4 HTN:5 Hyperlipidemia: 2

## Data Availability

Not applicable.

## References

[1] Thomas S.J., Moreira E.D., Kitchin N., Absalon J., Gurtman A., Lockhart S., Perez J.L., Pérez Marc G., Polack F.P., Zerbini C. (2021). Safety and Efficacy of the BNT162b2 mRNA Covid-19 Vaccine through 6 Months. N. Engl. J. Med..

[2] Baden L.R., El Sahly H.M., Essink B., Kotloff K., Frey S., Novak R., Diemert D., Spector S.A., Rouphael N., Creech C.B. (2021). Efficacy and Safety of the mRNA-1273 SARS-CoV-2 Vaccine. N. Engl. J. Med..

[3] Heath P.T., Galiza E.P., Baxter D.N., Boffito M., Browne D., Burns F., Chadwick D.R., Clark R., Cosgrove C., Galloway J. (2021). Safety and Efficacy of NVX-CoV2373 Covid-19 Vaccine. N. Engl. J. Med..

[4] Sadoff J., Gray G., Vandebosch A., Cárdenas V., Shukarev G., Grinsztejn B., Goepfert P.A., Truyers C., Van Dromme I., Spiessens B. (2022). Final Analysis of Efficacy and Safety of Single-Dose Ad26.COV2.S. N. Engl. J. Med..

[5] Voysey M., Clemens S.A.C., Madhi S.A., Weckx L.Y., Folegatti P.M., Aley P.K., Angus B., Baillie V.L., Barnabas S.L., Bhorat Q.E. (2021). Safety and efficacy of the ChAdOx1 nCoV-19 vaccine (AZD1222) against SARS-CoV-2: An interim analysis of four randomised controlled trials in Brazil, South Africa, and the UK. Lancet.

[6] World Health Organization COVID-19 Vaccines with WHO Emergency Use Listing. https://extranet.who.int/pqweb/vaccines/vaccinescovid-19-vaccine-eul-issued.

[7] Ng X.L., Betzler B.K., Ng S., Chee S.P., Rajamani L., Singhal A., Rousselot A., Pavesio C.E., Gupta V., de Smet M.D. (2022). The Eye of the Storm: COVID-19 Vaccination and the Eye. Ophthalmol. Ther..

[8] Lee Y.K., Huang Y.H. (2021). Ocular Manifestations after Receiving COVID-19 Vaccine: A Systematic Review. Vaccines.

[9] Lin T.P.H., Ko C.N., Zheng K., Lai K.H.W., Wong R.L.M., Lee A., Zhang S., Huang S.S., Wan K.H., Lam D.S.C. (2021). COVID-19: Update on Its Ocular Involvements, and Complications from Its Treatments and Vaccinations. Asia Pac. J. Ophthalmol..

[10] Ng X.L., Betzler B.K., Testi I., Ho S.L., Tien M., Ngo W.K., Zierhut M., Chee S.P., Gupta V., Pavesio C.E. (2021). Ocular Adverse Events After COVID-19 Vaccination. Ocul. Immunol. Inflamm..

[11] Song P., Xu Y., Zha M., Zhang Y., Rudan I. (2019). Global epidemiology of retinal vein occlusion: A systematic review and meta-analysis of prevalence, incidence, and risk factors. J. Glob. Health.

[12] Ip M., Hendrick A. (2018). Retinal Vein Occlusion Review. Asia Pac. J. Ophthalmol..

[13] Ophthalmologists T.R.C.o. (2015). Retinal Vein Occlusion (RVO) Guidelines.

[14] Klein R., Klein B.E., Moss S.E., Meuer S.M. (2000). The epidemiology of retinal vein occlusion: The Beaver Dam Eye Study. Trans. Am. Ophthalmol. Soc..

[15] Rogers S., McIntosh R.L., Cheung N., Lim L., Wang J.J., Mitchell P., Kowalski J.W., Nguyen H., Wong T.Y. (2010). The prevalence of retinal vein occlusion: Pooled data from population studies from the United States, Europe, Asia, and Australia. Ophthalmology.

[16] Bialasiewicz A.A., Farah-Diab M.S., Mebarki H.T. (2021). Central retinal vein occlusion occurring immediately after 2nd dose of mRNA SARS-CoV-2 vaccine. Int. Ophthalmol..

[17] Priluck A.Z., Arevalo J.F., Pandit R.R. (2022). Ischemic retinal events after COVID-19 vaccination. Am. J. Ophthalmol. Case Rep..

[18] Tanaka H., Nagasato D., Nakakura S., Tanabe H., Nagasawa T., Wakuda H., Imada Y., Mitamura Y., Tabuchi H. (2021). Exacerbation of branch retinal vein occlusion post SARS-CoV2 vaccination: Case reports. Medicine.

[19] Peters M.C., Cheng S.S.H., Sharma A., Moloney T.P. (2022). Retinal vein occlusion following COVID-19 vaccination. Clin. Exp. Ophthalmol..

[20] Sugihara K., Kono M., Tanito M. (2022). Branch Retinal Vein Occlusion after Messenger RNA-Based COVID-19 Vaccine. Case Rep. Ophthalmol..

[21] Ikegami Y., Numaga J., Okano N., Fukuda S., Yamamoto H., Terada Y. (2022). Combined central retinal artery and vein occlusion shortly after mRNA-SARS-CoV-2 vaccination. Qjm.

[22] Sacconi R., Simona F., Forte P., Querques G. (2022). Retinal Vein Occlusion Following Two Doses of mRNA-1237 (Moderna) Immunization for SARS-Cov-2: A Case Report. Ophthalmol. Ther..

[23] Park H.S., Byun Y., Byeon S.H., Kim S.S., Kim Y.J., Lee C.S. (2021). Retinal Hemorrhage after SARS-CoV-2 Vaccination. J. Clin. Med..

[24] Sonawane N.J., Yadav D., Kota A.R., Singh H.V. (2022). Central retinal vein occlusion post-COVID-19 vaccination. Indian. J. Ophthalmol..

[25] Lee S., Sankhala K.K., Bose S., Gallemore R.P. (2022). Combined Central Retinal Artery and Vein Occlusion with Ischemic Optic Neuropathy After COVID-19 Vaccination. Int. Med. Case. Rep. J..

[26] Romano D., Morescalchi F., Romano V., Semeraro F. (2022). COVID-19 AdenoviralVector Vaccine and Central Retinal Vein Occlusion. Ocul. Immunol. Inflamm..

[27] Chen Y.C. (2022). Combined central retinal artery occlusion and vein occlusion with exudative retinal detachment following COVID-19 vaccination. Kaohsiung J. Med. Sci..

[28] Vujosevic S., Limoli C., Romano S., Vitale L., Villani E., Nucci P. (2022). Retinal vascular occlusion and SARS-CoV-2 vaccination. Graefes Arch. Clin. Exp. Ophthalmol..

[29] Dutta Majumder P., Prakash V.J. (2022). Retinal venous occlusion following COVID-19 vaccination: Report of a case after third dose and review of the literature. Indian J. Ophthalmol..

[30] Pur D.R., Catherine Danielle Bursztyn L.L., Iordanous Y. (2022). Branch retinal vein occlusion in a healthy young man following mRNA COVID-19 vaccination. Am. J. Ophthalmol. Case Rep..

[31] Endo B., Bahamon S., Martínez-Pulgarín D.F. (2021). Central retinal vein occlusion after mRNA SARS-CoV-2 vaccination: A case report. Indian J. Ophthalmol..

[32] Shah P.P., Gelnick S., Jonisch J., Verma R. (2021). Central Retinal Vein Occlusion Following BNT162b2 (Pfizer-BioNTech) COVID-19 Messenger RNA Vaccine. Retin. Cases Brief Rep..

[33] Sodhi P.K., Yadav A., Sharma B., Sharma A., Kumar P. (2022). Central Retinal Vein Occlusion Following the First Dose of COVID Vaccine. Cureus.

[34] Takacs A., Ecsedy M., Nagy Z.Z. (2022). Possible COVID-19 MRNA Vaccine-Induced Case of Unilateral Central Retinal Vein Occlusion. Ocul. Immunol. Inflamm..

[35] Karageorgiou G., Chronopoulou K., Georgalas I., Kandarakis S., Tservakis I., Petrou P. (2022). Branch retinal vein occlusion following ChAdOx1 nCoV-19 (Oxford-AstraZeneca) vaccine. Eur. J. Ophthalmol..

[36] Nangia P., Prakash V.J., Dutta Majumder P. (2022). Retinal venous occlusion in a child following Corbevax COVID-19 vaccination. Indian J. Ophthalmol..

[37] Tanaka H., Nagasato D., Nakakura S., Nagasawa T., Wakuda H., Kurusu A., Mitamura Y., Tabuchi H. (2022). Branch retinal vein occlusion post severe acute respiratory syndrome coronavirus 2 vaccination. Taiwan J. Ophthalmol..

[38] Cunningham E.T., Moorthy R.S., Agarwal M., Smit D.P., Zierhut M. (2022). Ocular Complications Following COVID-19 Vaccination—Coincidence, Correlation, or Causation?. Ocul. Immunol. Inflamm..

[39] Thiele T., Ulm L., Holtfreter S., Schönborn L., Kuhn S.O., Scheer C., Warkentin T.E., Bröker B.M., Becker K., Aurich K. (2021). Frequency of positive anti-PF4/polyanion antibody tests after COVID-19 vaccination with ChAdOx1 nCoV-19 and BNT162b2. Blood.

